# Preventive electroacupuncture ameliorates D-galactose-induced Alzheimer’s disease-like inflammation and memory deficits, probably via modulating the microbiota–gut–brain axis

**DOI:** 10.22038/ijbms.2021.49147.11256

**Published:** 2021-03

**Authors:** Chuan He, Zhong-Sheng Huang, Chao-Chao Yu, Xue-Song Wang, Tao Jiang, Miao Wu, Li-Hong Kong

**Affiliations:** 1 College of Acupuncture and Orthopedics, Hubei University of Chinese Medicine, Wuhan, Hubei, China; 2 Hubei Provincial Collaborative Innovation Center of Preventive Treatment by Acupuncture and Moxibustion, Hubei University of Chinese Medicine, Wuhan, Hubei, China; 3 Department of Tuina, Shenzhen Traditional Chinese Medicine Hospital; 4 The 4^th^ Clinical College of Guangzhou University of Chinese Medicine, Shenzhen, China; 5 Hubei Provincial Hospital of TCM, Wuhan, Hubei, China; 6 Hubei Province Academy of Traditional Chinese Medicine, Wuhan, Hubei, China

**Keywords:** Aging, Alzheimer’s disease, Electroacupuncture, Microbiota-gut-brain axis, NF-κB, TLR4

## Abstract

**Objective(s):**

We aimed to observe the effects of preventive electroacupuncture (EA) on the microbiota–gut–brain axis and spatial learning and memory deficits and to investigate the possible mechanism using D-galactose (D-gal)-induced aging rats.

**Materials and Methods:**

D-gal was intraperitoneally injected to establish the aging model. We used Morris water maze to detect spatial learning and memory function of rats. RT-PCR was applied to test targeted gut microbes. The expression of zonula occludens-1 (ZO-1) and Toll-like receptor 4 (TLR4)/nuclear factor (NF)-κB pathway proteins were detected by Western blotting. ELISA was employed to evaluate the level of lipopolysaccharides (LPS), diamine oxidase (DAO) and S-100β. Additionally, we observed ionized calcium-binding adapter molecule-1 (Iba-1) expression in the hippocampal CA1 area by immunofluorescence.

**Results:**

Morris water maze test showed decreased mean escape latency and increased target quadrant time after EA treatment. The gut microbiota composition has been modified in EA treated rats. Molecular examination indicated that expression of ZO-1 was improved and the the concentration of LPS in blood and hippocampus were reduced in EA treated rats. Further, we observed an inhibition of activated microglia and TLR4/NF-κB pathway in EA groups.

**Conclusion:**

Preventive EA may alleviate the impairments of the microbiota–gut–brain axis and spatial learning and memory in aging, and the mechanism may be related to the inhibition of TLR4/NF-kB signaling pathway. The combination of acupoints GV20 and ST36 can enhance the therapeutic effect in aging rats.

## Introduction

Alzheimer’s disease (AD) is a notorious neurodegenerative disorder characterized by cognitive impairments and memory loss leading to dementia (1). Aging is one of the prominent causes of AD. As the worldwide population ages, the incidence of AD continues to rise significantly. More than 132 million people are expected to be affected by 2050 (1). Unfortunately, there are still no effective therapeutic methods that can stop the progression of AD. Therefore, it is of great significance to find effective methods for early prevention of AD.

A rapidly growing number of scientific reports in recent years has suggested the dysbiosis of gut microbiota might be the source of AD (2). The human gastrointestinal tract is inhabited by trillions of bacteria, belonging to more than 15,000 species (3). The rational structure of the gut microbiota is known to be beneficial for the host’s health, while microbiota dysbiosis increases the host’s susceptibility to diseases such as inflammatory bowel disease, metabolic diseases, respiratory diseases, autoimmune diseases, and neurodegenerative diseases (4, 5). The microbiota–gut–brain axis, proposed to be the bidirectional communication pathway between the brain and gut, is recognized to be connected through cytokine, immunological, hormonal, and neuronal signals (6, 7). Studies have suggested that aging-induced dysbiosis of the gut microbiota correlated with chronic inflammation, which can also produce abundant inflammatory response substances such as amyloids, lipopolysaccharides (LPS), and various microbial exudates (8, 9). These powerful proinflammatory substances can cross the damaged biological barriers during aging, which might initiate neuro-inflammation and neurodegeneration.

LPS is the main component of the cell wall of Gram-negative bacteria and has a strong inflammatory effect on the human central nervous system (CNS) (10). Aging-induced gut microbiota dysbiosis is often accompanied by excretion of enormous quantities of LPS. Studies have reported increased LPS level in brain of AD patients (11). Elevated LPS content in the plasma and brain has also been found in aging mice (12). Briefly, LPS produced by gut microbiota dysbiosis during aging can pass through the damaged intestinal barrier and blood brain barrier and induce neuro-inflammation, eventually leading to neurodegeneration (13).

Microglia is one of the important immune cells in CNS, which can be activated by LPS through the CD14/TLR4 receptor complex. The downstream signal transduction of this receptor complex activates the Toll-like receptor 4 (TLR4)/nuclear factor-kB (NF-kB) signaling pathway, which leads to the production of inflammatory cytokines and thus neuro-inflammatory response (14-16). An acute, self-limiting neuro-inflammatory response is beneficial to reduce the damage of neuropathological stimulants to neurons (17). However, during the aging process, continuous stimulation by LPS and other bacterial metabolites puts the microglia in a persistent state of activation, which leads to a chronic state of self-reinforcing inflammation (18). Collectively, these studies imply that LPS produced by the impaired gut microbiota might induce neuro-inflammation, which causes neuronal death and cognitive impairment in the aging process.

Researches have shown the advantageous effects of needle therapy in alleviating cognitive impairment in AD (19, 20). Studies have also confirmed that acupuncture could regulate the gut microbiota (21). Based on previous studies, we further explored whether simultaneous EA treatment, under the guidance of the theory of “disease prevention by acupuncture ”in Chinese medicine, could reduce learning and memory shortfalls by regulating gut microbiota LPS-induced neuro-inflammation in aging.

## Materials and Methods

***Animals and groups***

Sprague Dawley rats purchased from the Hubei Experimental Animal Research Center were used in this study, with a weight 360±20 g. The rats were kept in temperature-and light-regulated cage rooms with 12/12 hr light/dark cycle at the 20±2 °C temperature. Food and water were provided *ad libitum.* Rats were assigned into control group, model group, EA(GV20) group, EA(ST36) group and EA(GV20+ ST36) group, 12 rats in each group. All animal experiments carried out were under the guidance of Ethical Committee Acts of Hubei University of Chinese Medicine. In the model, EA(GV20), EA(ST36) and EA(GV20+ST36) groups, 120 mg D-galactose was intraperitoneally injected to rats daily for 8 weeks (22), while the control group was not injected.

***EA treatment***

 Our early study described the process of EA treatment (23). The rats in EA groups were treated with EA after daily D-gal injection for eight weeks. GV20 also called Baihui acupoint was located at the center of the parietal bone and ST36 known as Zusanli acupoint which located at the notch 4 mm lateral to the anterior tuber point of the tibia.

In the process of EA treatment, the rats were fixed on a special table with specially made soft clothes in prone position, and then the disposable acupuncture needles were inserted into the acupoints and connected to the EA apparatus. GV20 point was punctured at an angle of 15 degrees for 2 mm depth and ST36 point was punctured perpendicularly for 4 mm. Continuous wave with frequency of 50 Hz and the intensity of 1 mA was selected in EA treatment for 20 minutes in accordance with our previous study (23). There were no interventions for the control and model group rats.

***Morris water maze test***

Our previous research described the experimental process of a water maze in detail (23). The Watermaze 2.0 software (Chengdu Taimeng Technology Co., Ltd., Chengdu, China) was used to record data and swimming path.

***Western blot assay***

Western blot assy was used to test the expression of ZO-1, E. coli LPS, TLR4, Phospho-NF-κB p65, IL-6, TNF-α and IL-1β with the following primary antibodies: ZO-1(ab221547, 1:500, Abcam); *E. coli *LPS(ab35654, 1:1000, Abcam), TLR4(19811-1-AP, 1:1000, Proteintech, Wuhan, China); Phospho-NF-κB p65(#3033, 1:500, Cell Signaling Technology);TNF-α(ab66579, 1:500, Abcam); IL-6 (ab208113, 1:500, Abcam); IL-1β (bs-0812R, 1:500, Bioss, Inc, China). The process was performed in strict accordance with the manufacturer’s instructions.

***Immunofluorescence staining***

Rat brain paraffin sections were incubated with primary antibody Iba-1 (ab5076, 1:100, Abcam) overnight after antigen retrieval. The samples were then washed and incubated with cyanine 3 (CY3) at 1:50. After washed 3 times, 4′,6-diamidino-2-phenylindole (DAPI) was used to stain the nuclei and glass cover slips were covered through fluorescent mounting medium over the slides. Photomicrographs were acquired through Laser Scanning Confocal Microscope (Leica, Type:TCS SP8, Germany).

***Enzyme-linked immunosorbent assay***

The concentrations of diamine oxidase (DAO), S-100β, LPS and inflammatory factors in freshly collected serum samples were measured using ELISA kits (RA20028 RA20420 RA20650 RA20035 RA20020 RA20607; Bioswamp, Wuhan, China) following standard instructions. N=6 rats for each experimental group.

***Real-time RT-PCR***

Bacterial DNA of the intestinal mucosal scrapings was extracted using Tissue Genomic DNA Extraction Kit (EP007, ELK Biotechnology, Wuhan, China) by centrifugation for 1 min at 12000 g. The cDNA was generated using cDNA Synthesis Kit (EQ003, ELK Biotechnology, Wuhan, China) with 2 μg RNA after confirming the RNA concentration and assessment of purity. RT-PCR was done through SYBR Green PCR SuperMixE(Q001 ELK Biotechnology, Wuhan, China) and q-RT-PCR machine from Life Technologies. Four targeted microbes for research were selected including* Lactobacillus, Bifidobacterium, Bacteroides fragilis*, and *Escherichia coli*. Primers were used as shown in Table 1. The process of incubation: 95 °C for 3 min, followed by 40 cycles of 95 °C for 10 sec, and 58 °C for 30 sec, then 72 °C for 30 sec. The relative expression level of the targeted genes was calculated using 2^–ΔΔCt^ method.

***Statistical analysi***

GraphPad Prism 7.01 was used to perform statistical analysis and the data were expressed as the mean±SD. One-way ANOVA followed by Tukey *post hoc* test was used to indicate significant difference between the groups. The value of *P* less than 0.05 was considered statistically significant.

## Results

***Preventive electroacupuncture improved spatial learning and memory impairments in aging rats***

**Figure 1:**
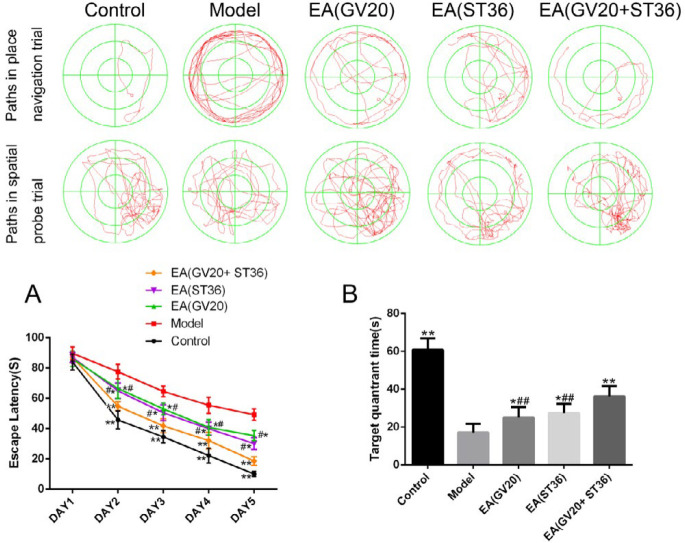
Results of the Morris water maze test

As presented by Figure 1, the mean escape latency in model group rats was significantly increased when compared with control (*P*<0.01), while the mean escape latency was decreased in EA groups compared with the model group (*P*<0.01). Interestingly, the mean escape latency of the EA(GV20+ST36) group was significantly decreased compared with the EA(GV20) and EA(ST36) groups (*P*<0.01, Figure 1A). We tested memory impairment by removing the hidden platform. We found that the rats in model group spent less time in the target quadrant compared with the control group (*P*<0.01), and the rats in EA treatment groups spent more time in target quadrant compared with the model (*P*<0.01). The rats in EA(GV20+ST36) group explored in the quadrant of the original platform for a longer time compared with the EA(GV20) and EA(ST36) groups (*P*<0.01, Figure 1B).

***Preventive electroacupuncture modified microbiota composition in aging rats***

**Figure 2:**
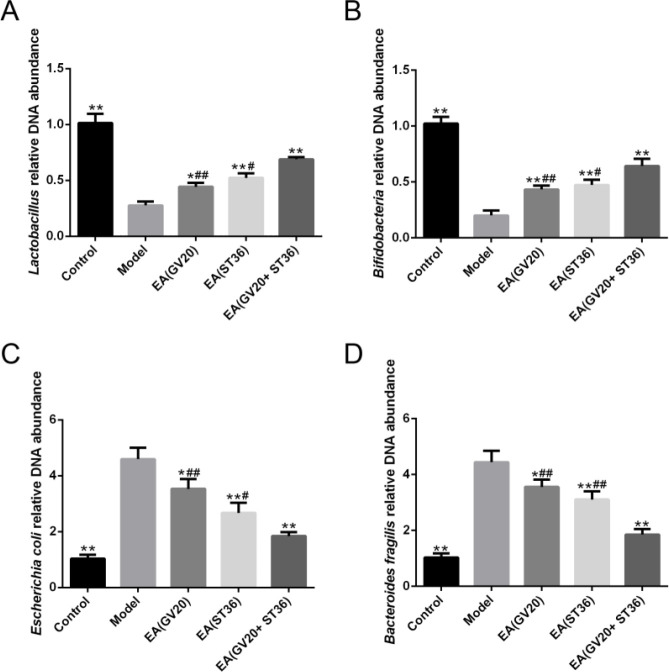
Preventive electroacupuncture treatment modified targeted gut microbiota composition. (A, B, C, D) Relative DNA abundance of *Lactobacillus*, *Bifidobacterium*, *Escherichia coli*, and *Bacteroides fragilis*. The data are expressed as mean±SD (n=6). *P<0.05, **P<0.01 vs. model; #*P*<0.05, ##*P*<0.01 vs. EA(GV20+ST36)

Real-time PCR was applied to assess the effect of EA on the regulation of targeted gut microbiota. As illustrated in Figure 2A–D, the relative DNA abundance of *Lactobacillus* and *Bifidobacterium* was significantly down-regulated in the model group than control group (*P*<0.01), and *E. coli* and *Bacteroides fragilis* was more highly expressed in the model group than control group (*P*<0.01). The EA groups showed higher relative DNA abundance of *Lactobacillus* and *Bifidobacterium* (*P*<0.01) and lower DNA abundance of *E. coli* and *B. fragilis* (*P*<0.01) when compared with the model group. Moreover, EA(GV20+ST36) could increase the relative DNA abundance of *Lactobacillus* and *Bifidobacterium* and reduce* E. coli* and* B. fragilis* to a greater extent than the other two preventive EA conditions (*P*<0.01).

***Preventive electroacupuncture restored intestinal mucosa barrier function and reduced serum LPS level in aging rats***

**Figure 3:**
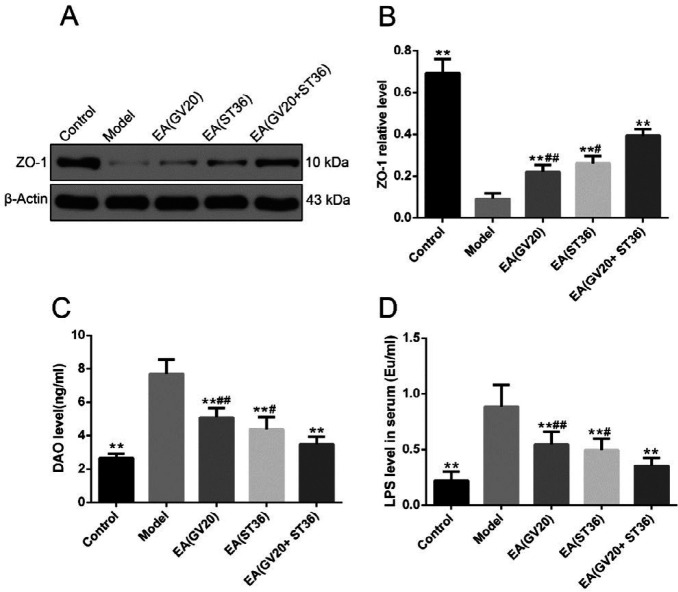
Preventive electroacupuncture restored intestinal mucosa barrier function and reduced LPS level in serum. (A, B) The ZO-1 protein expression in the intestine was measured by Western blotting and quantitatively analyzed. (C) DAO level in serum. (D) LPS level in serum. The data are expressed as mean±SD (n=6). **P<0.01 vs. model, #*P*<0.05,# #*P*<0.01 vs. EA(GV20+ST36)

Epithelial tight junction protein zonula occludens-1 (ZO-1) is one of the most important components of the intestinal mucosal barrier (24). Serum diamine oxidase (DAO) can be used as a serological marker in evaluating intestinal injury and its increasing concentration in serum indicates increased intestinal epithelial permeability or damage to intestinal barrier function (25). Therefore, ZO-1 and DAO levels were evaluated to test the intestinal mucosal barrier function by Western blotting and ELISA, respectively. As shown in Figure 3B, the ZO-1 expression was down-regulated in the model group compared with the control group (*P* <0.01), which was significantly increased in EA groups compared with the model group (*P*<0.01). Moreover, EA(GV20+ST36) increased the protein expression level of ZO-1 to a greater extent than the other two preventive EA conditions (*P*<0.01). As shown in Figure 3C and 3D, the concentrations of DAO and LPS were remarkably elevated in the model group than the control group (*P*<0.01), and preventive EA significantly reduced DAO and LPS concentrations compared with the model group (*P*<0.05 or *P*<0.01). Moreover, EA(GV20+ST36) decreased DAO and LPS concentrations to a greater extent than the other two preventive EA conditions (*P*<0.05 or *P*<0.01). In general, these data suggested that EA treatment improved intestinal barrier injury and prevented LPS from entering the blood from the gut.

***Preventive electroacupuncture restored BBB function and reduced the LPS level in the brain of aging rats***

**Figure 4:**
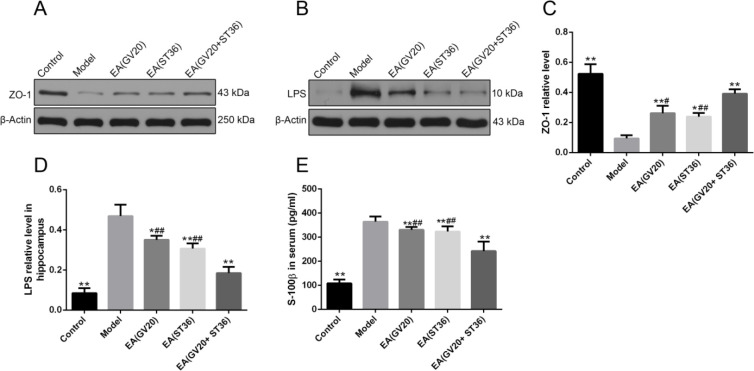
Preventive electroacupuncture restored BBB function and reduced LPS level in hippocampus of aging rats. (A–D) Western blot was used to test ZO-1 and LPS expression level in the hippocampus. (E) S-100β level in serum. The data are expressed as mean±SD (n=6). *P<0.05, **P<0.01 vs. model, #*P*<0.05, ##P < 0.01 vs. EA(GV20+ST36)

The tight junction proteins are indispensable part of BBB (26), so we assessed the expression of tight junction protein ZO-1 with western blotting. Serum S-100β was reported to be a marker of leaky BBB (27); therefore, S-100β level in serum was detected by ELISA. As presented in Figure 4C, the protein level of ZO-1 was significantly decreased in the model group than control group (*P*<0.01), which was significantly increased after EA treatment compared with the model group (*P*<0.01), and EA(GV20+ST36) increased the protein level of ZO-1 to a greater extent than the other two preventive EA conditions (*P*<0.01). The concentration of S-100β was remarkably increased in the model group than the control group (*P*<0.01). Preventive EA significantly decreased S-100β concentrations compared with the model group (*P*<0.01). Besides, EA(GV20+ST36) decreased the S-100β concentration to a greater extent than the other two preventive EA conditions (*P*<0.01, Figure 4E). As shown in Figure 4D, the remarkably higher LPS level in the hippocampus was observed in model group than in the control group (*P*<0.01), which was effectively decreased by EA intervention (*P*<0.05 or *P*<0.01). These results suggested that EA treatment alleviated BBB injury and reduced the LPS level in the brain of aging rats.

***Preventive electroacupuncture suppressed the activated microglia in aging rats***

**Figure 5:**
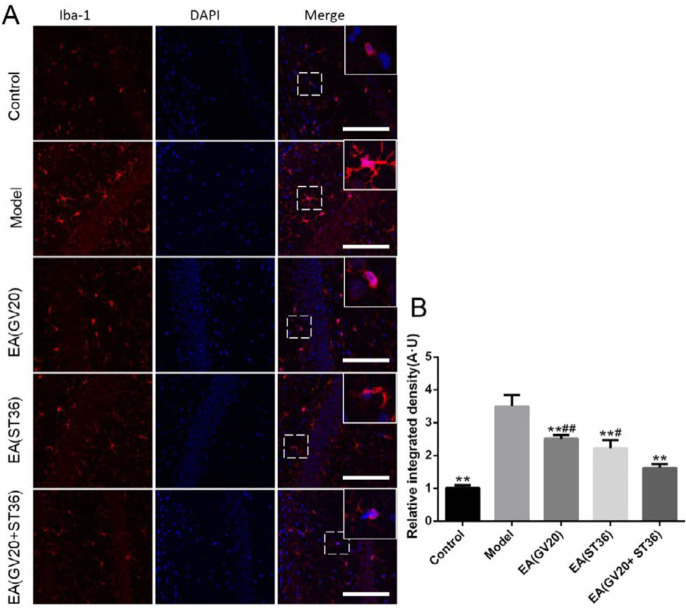
Preventive electroacupuncture alleviated the activation of microglia in aging rats. (A) Photomicrographs of microglia (Iba-1-positive cells) in the hippocampal CA1 area. Scale bar, 25 μm. (B) Quantitative photomicrograph analysis of relative integrated density of Iba-1. The data are expressed as mean±SD (n=5). **P<0.01 vs. model, #*P*<0.05, ##*P*<0.01 vs. EA(GV20+ST36)

LPS is a classical pathogen-associated molecular pattern (PAMP) that can activate microglia. Activated microglia are one of the major factors in the development of neuro-inflammation (17). To evaluate the effect of EA on microglia, Iba-1, a specific marker of activated microglia, was detected. As illustrated in Figure 5, the significantly up-regulated immunoreactivity of Iba-1 has been observed in the hippocampal CA1 area of model group rats compared with control (*P*<0.05). Such immunoreactivity was reduced by EA treatment, and EA(GV20+ ST36) outperformed treatment with single acupoint EA(GV20) or EA(ST36) individually (*P*<0.05 or *P*<0.01).

***Preventive electroacupuncture inhibited the TLR4/NF-κB signaling pathway in aging rats***

**Figure 6:**
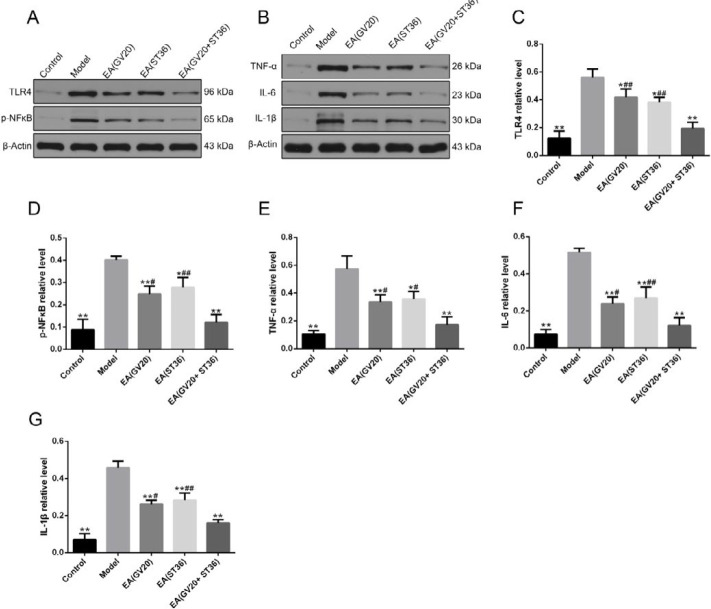
Preventive electroacupuncture inhibited the TLR4/NF-κB signaling pathway in aging rats. (A-G) Western blotting was used to detect the protein levels of TLR4, P-NF-κB p65, TNF-α, IL-6, and IL-1β in the hippocampus. The data are expressed as mean±SD (n=6). *P< 0.05, **P<0.01 vs. model, #*P*<0.05, ##*P*<0.01 vs. EA(GV20+ST36)

Toll-like receptors (TLRs) that expressed on microglia play important roles in recognizing LPS and other body pathogens (28). LPS can activate the TLR4/NF-κB pathway, which then causes the production of downstream pro-inflammatory cytokines and neurodegeneration (29). Therefore, we further detected the effect of preventive EA on the TLR4/NF-κB pathway and downstream proinflammatory cytokines. As shown in Figure 6, we found significantly increased protein expression of TLR4, P-NF-κB p65, TNF-α, IL-6, and IL-1β in model group rats than control (*P*<0.01), which could be inhibited by EA treatment compared with the model group (*P*<0.05 or *P*<0.01). Our results indicated that preventive EA treatment can significantly inhibit TLR4/NF-kB signaling pathway thus alleviating neuro-inflammation.

## Discussion

Our research demonstrated that EA treatment effectively restored the learning and memory impairments of D-gal-induced aging rats, accompanied by the inhibition of the TLR4/NF-kB signaling pathway and neuro-inflammation. In addition, EA treatment can also regulate the gut microbiota and inhibit increased LPS levels in serum and the hippocampus. Moreover, the compromised intestinal barrier and BBB are restored by EA treatment.

D-gal-induced aging rats are widely used for studying the process of aging. Studies have shown that systemic long-term use of D-galactose can lead to memory deficits, neuronal degeneration and apoptosis, increased oxidative stress, and neuro-inflammation in animals (30, 31). Moreover, our previous study observed memory impairments and damagement of synapse and neuronal microtubule in D-gal-induced aging rats.(32). In the present study, impaired spatial reasoning and memory ability were observed in the model group, which is consistent with previous studies. Moreover, EA treatment can remarkably improve the cognitive function of aging rats in the Morris water maze tests.

Meanwhile, EA treatment obviously alleviated neuro-inflammation in EA groups compared with the model group. We observed activated Iba-1-positive cells and elevated protein expression of TNF-α, IL-6, and IL-1β in the hippocampus of aging rats, suggesting the activation of microglia and inflammatory responses. Persistent activation of microglia during aging can release a large quantity of inflammatory cytokines in response to injurious stimuli, thus leading to the damage of neurons and neurodegeneration (17, 33). Recent studies reported that EA treatment can ameliorated neuro-inflammation thus cognitive impairment in AD mice (34, 35). The inhibition of microglia activation after EA treatment suggests that EA at the GV 20 and ST 36 acupoints could effectively alleviate neuro-inflammation, as a result, alleviating cognitive dysfunction.

Accumulating evidence supports the idea that the dysfunction of microbiota–gut–brain axis is closely related to the progression of neurodegenerative diseases like AD (36, 37). For instance, bacteria and their noxious exudates found in the brains of AD patients, indicating that microbes may contribute to AD-associated neuro-inflammation (38, 39). Intestinal inflammation has also been found to have a clear connection with microglial neuro-inflammation (40-43). The aging process is characterized by a decrease in the biodiversity of the intestinal flora, such as increased abundance of the Enterobacteriaceae family as well as a reduced number of *Bifidobacterium* species (44-46). The decrease of probiotics and the increase of inflammatory bacteria seem to be the characteristics of intestinal flora imbalance during aging (47). Such alteration of intestinal flora can cause intestinal inflammation and intestinal barrier dysfunction as well as increased permeability of the gut, which contributes the entry of gut bacteria-derived harmful substances, such as LPS, into the bloodstream and the brain (48, 49). The *B. fragilis *and *E. coli *can release abundant pro-inflammatory LPS in human gut (50). Microbiota modulation is used as a potential therapeutic intervention in AD. Numerous studies have suggested that the administration of probiotics such as *Bifidobacterium bifidum,* *Lactobacillus acidophilus, *and *Bifidobacterium lactis* exerts a neuroprotective effect by enhancing intestinal barrier and BBB integrity thus reducing neuro-inflammation (51-53). To clarify the mechanism of electroacupuncture in the treatment of cognitive dysfunction in D-gal-induced aging rats, the selected intestinal bacteria, including those of *Lactobacillus*, *Bifidobacterium*, *B. fragilis*, and *E. coli *were tested with real-time PCR. We found that *Lactobacillus* and *Bifidobacterium* showed up-regulated relative DNA abundances while decreased abundance of *Bacteroides fragilis *and *E. coli* after EA treatment. This is consistent with previous studies showing that moxibustion at acupoints can regulate the intestinal flora in other disease models (54, 55). These findings suggest that EA treatment may improve cognitive dysfunction by influencing gut microbiota composition.

Indeed, both the intestinal barrier and the BBB are weakened during aging, and the malfunction of these physiological barriers may lead to systemic inflammation and chronic neuro-inflammation, which is thought to contribute to the pathogenesis of AD (56). Decreased BBB permeability as well as the expression of claudin-5, occludin, and ZO-1 proteins has been confirmed in aging animals (57, 58). Moreover, serum levels of S100B, a commonly used indicator of BBB permeability, has also been found to be significantly increased in aging mice (59). In aging, the up-regulation of the inflammatory cytokines can remodel tight junctions, which leads to damaged intestinal barrier and BBB integrity (58, 60). Cytokines produced by peripheral inflammation may activate the microglia and induce secondary neuro-inflammation (61). In the present study, the intestinal barrier and BBB integrity were tested by western blotting and ELISA in aged rats. We found that the protein expression level of ZO-1 in the brain and intestine were significantly decreased and the concentrations of S-100β and DAO in serum were remarkably elevated in the model group compared with control. Moreover, EA treatment can also increase the expression of ZO-1 protein and reduce the S-100β and DAO levels. Furthermore, ELISA assay was used to examine inflammatory responses in systemic circulation in aged rats. Our results showed that D-gal injection significantly up-regulated levels of IL-6, IL-1β, and TNF-a, which could be down-regulated through EA treatment. Collectively, our results indicate that the therapeutic effects of EA on the CNS may be related to its modulation on dysfunctional microbiota–gut–brain axis as well as an inhibition of peripheral inflammation in aging rats.

As mentioned before, LPS is a well known pathogen-associated molecular pattern which can induce neuro-inflammation via the activation of TLR4/NF-kB signaling pathway. In this study, elevated LPS contents in the plasma and brain as well as activated TLR4/NF-kB pathway have been observed in model group rats compared with controls. Notably, TLR4/NF-kB signaling can be significantly inhibited by EA intervention in aging rats and a previous study also indicating that EA treatment at ST36 can inhibit TLR4/NF-κB signaling thus alleviating LPS-induced inflammation in rats (62). In summary, our results suggested that inflammatory substances produced by intestinal flora imbalance could induce subsequent inflammatory response in the brain and EA treatment may protect the CNS by inhibiting TLR4/NF-κB signaling pathway. 

Our results also indicated the combination of GV20 and ST36 outperformed treatment with GV20 or ST36 individually in aging rats. The acupoint combination is indispensable to improve the clinical efficacy of acupuncture in traditional Chinese medicine theory. GV20 is the most commonly used acupoint to treat neuropsychiatric disorders, while ST36 is the most commonly chosen acupoint to treat gastrointestinal disease. Studies have indicated that acupuncture at ST36 has the effect of protecting the intestinal barrier and regulating the gut microbiota in rats (63). Therefore, it is necessary to combine these two points to treat cognitive decline in aging rats.

Herein, we observed that preventive EA may rescue cognitive dysfunction in D-gal-Induced aging rats via modulating the dysbiosis of gut microbiota and impaired microbiota-gut-brain axis. And the neuroprotective effect of EA may associate with the down-regulation of TLR4/NF-κB signaling pathway. There are also some limitations in our study. For instance, only four selective gut microbiota profiles were detected, and future studies can analyze gut microbiota composition by 16S rRNA gene sequencing to further observe the overall changes of gut microbiota composition. Besides, it can be more persuading to also examine the changes of intestinal boundary and BBB utilizing transmission electron microscope to display more visualized ultrastructure changes.

**Table 1:** The sequences of primers used for amplification of selective intestinal microbiota

**Microorganism**		**Base sequence**
*Lactobacillus*	Primer F	5'-ACGGGAGGCAGCAGTAGGGA-3'
	Primer R	5'-AGCCGTGACTTTCTGGTTGATT-3'
*Bifidobacterium*	Primer F	5'-GATTCTGGCTCAGGATGAACGC-3'
	Primer R	5'-CTGATAGGACGCGACCCCAT-3'
*Bacteroides fragilis*	Primer F	5'-GGATACATCAGCTGGGTTGTAG-3'
	Primer R	5'-GCGAACTCGGTTTATGCAGT-3'
Escherichia coli	Primer F	5'-CATGCCGCGTGTATGAAGAA-3'
	Primer R	5'-CGGGTAACGTCAATGAGCAAA-3'

(A) The escape latency in the hidden platform trial. (B) The target quadrant time. The data are expressed as mean±SD (n=12). *P<0.05, **P<0.01 vs. model; #*P*<0.05, ##*P*<0.01 vs. EA(GV20+ST36)

## Conclusion

Our results show that preventive EA treatment during aging process can restore the learning and memory impairments of aging rats by regulating gut microbiota, and the impaired microbiota-gut-brain axis. The neuroprotective effect of EA is probably related with the down-regulation of TLR4/NF-κB signaling pathway. The combination of acupoints GV20 and ST36 can enhance therapeutical effect in D-gal-induced aging rats.
